# Incidence of GLP‐1 receptor agonist use by women of reproductive age attending general practices in Australia, 2011–2022: a retrospective open cohort study

**DOI:** 10.5694/mja2.70026

**Published:** 2025-09-01

**Authors:** Kailash Thapaliya, Arianne Sweeting, Kirsten I Black, Amanda Poprzeczny, Danielle Mazza, Luke E Grzeskowiak

**Affiliations:** ^1^ Flinders University Adelaide SA; ^2^ South Australian Health and Medical Research Institute Adelaide SA; ^3^ Royal Prince Alfred Hospital Sydney NSW; ^4^ The University of Sydney Sydney NSW; ^5^ Sydney Local Area Health District Sydney NSW; ^6^ The University of Adelaide Adelaide SA; ^7^ Women's and Children's Hospital Adelaide Adelaide SA; ^8^ Monash University Melbourne VIC; ^9^ SPHERE NHMRC Centre of Research Excellence Monash University Melbourne VIC; ^10^ SA Pharmacy SA Health Adelaide SA

**Keywords:** Pharmacoepidemiology, Reproduction, Diabetes mellitus, type 2, Contraception

## Abstract

**Objective:**

To examine longitudinal changes in the initial prescribing of glucagon‐like peptide 1 (GLP‐1) receptor agonists for women of reproductive age in Australia; to determine whether contraception recommendations are being followed; and to estimate the frequency of pregnancy among women using GLP‐1 receptor agonists.

**Study design:**

Retrospective open cohort study; analysis of MedicineInsight general practice data.

**Setting, participants:**

Women aged 18–49 years who visited participating general practices three or more times during the study period (1 January 2011 – 31 July 2022).

**Main outcome measures:**

Age‐standardised incidence of initial GLP‐1 receptor agonist prescribing, by year and type 2 diabetes status; proportion of women using highly effective contraception at the time of GLP‐1 receptor agonist initiation (contraception overlap); age‐standardised incidence of pregnancy within six months of the first prescribing of GLP‐1 receptor agonists.

**Results:**

Of 1 635 684 women aged 18–49 years, 18 010 (1.1%) were first prescribed GLP‐1 receptor agonists during 2011–2022, of whom 3739 (20.8%) had type 2 diabetes. The age‐standardised incidence of GLP‐1 receptor agonist prescribing for women with type 2 diabetes increased from 13.0 per 1000 women in 2011 to 88.5 per 1000 women in 2022; for women without type 2 diabetes, it increased from 0 to 14.9 per 1000 women. Of the 6293 women first prescribed GLP‐1 receptor agonists during 2022, 6954 (90.5%) did not have type 2 diabetes. Contraception overlap with first prescribing of GLP‐1 receptor agonists was determined for 3825 women (21.2%). Pregnancies within six months of GLP‐1 receptor agonist prescribing were documented for 232 of 10 781 women for whom at least six months of follow‐up data were available.

**Conclusions:**

The prescribing of GLP‐1 receptor agonists for women of reproductive age is increasing in Australia, and most prescriptions are for women not diagnosed with type 2 diabetes. Fewer than one in four women are using contraception at the time of treatment initiation, and a considerable number are pregnant within six months of the initial prescribing of GLP‐1 receptor agonists. Further evidence and guidelines are needed to support the safe and effective use of GLP‐1 receptor agonists by women of reproductive age.

**The known**: Glucagon‐like peptide 1 (GLP‐1) receptor agonists are increasingly popular for managing type 2 diabetes and weight loss. Concurrent contraception is recommended for women of child‐bearing age because of safety concerns raised by animal studies.

**The new**: The prescribing of GLP‐1 receptor agonists for women of reproductive age has increased rapidly in recent years, particularly for managing weight loss. The rate of concurrent contraception is low, and pregnancy within six months of initiating treatment not infrequent.

**The implications**: Given the increasing use of GLP‐1 receptor agonists and low rate of contraception overlap among women using them, their potential effects on the fetus are of concern.

Glucagon‐like peptide‐1 (GLP‐1) receptor agonists mimic the effects of the naturally occurring hormone GLP‐1; produced in the body during eating, it stimulates insulin secretion from the pancreas to reduce blood glucose levels.^1^ Initially promoted for managing type 2 diabetes,^2^ the effectiveness of GLP‐1 receptor agonists for suppressing appetite and promoting weight loss has led to their widespread promotion and off‐label use for managing obesity.^1^, ^3^ Increased prescribing has led to supply shortages in Australia, as reported by the Therapeutic Goods Administration.^4^

In Australia, about 1% of women of reproductive age have type 2 diabetes,^5^, ^6^ and more than 40% of these women also have overweight or obesity.^7^ Further, 1% of pregnancies are affected by type 2 diabetes and more than 50% by overweight or obesity,^8^ each of which is associated with increased risk of adverse pregnancy outcomes. Promoting adequate contraception use and effective pre‐conception care are consequently important for women with diabetes or overweight, particularly as about 40% of pregnancies in Australia are unplanned.^9^, ^10^

GLP‐1 receptor agonists can be highly effective treatments for women of reproductive age with diabetes and obesity, but concerns about their safety during pregnancy have been expressed. Animal studies indicate that GLP‐1 receptor agonist exposure during pregnancy leads to reduced fetal weight or growth, delayed ossification and skeletal variants, and reduced maternal weight gain.^11^ The only study in humans found no association between GLP‐1 receptor agonist exposure and congenital malformations, but other potential adverse pregnancy outcomes were not examined.^12^ The Medicines and Healthcare products Regulatory Agency in the United Kingdom therefore recommends that GLP‐1 receptor agonists not be used during pregnancy and that women of reproductive age prescribed these agents should use effective contraception.^13^ Anecdotal reports of unplanned pregnancies in women using GLP‐1 receptor agonists are nevertheless increasing, but robust data on their use, contraception use, and pregnancies among Australian women of reproductive age are not available.

We therefore investigated longitudinal changes in the initial prescribing of GLP‐1 receptor agonists for women of reproductive age in Australia, analysing data from a large national general practice research database. We sought to determine whether contraception recommendations are being followed, and to estimate the frequency of pregnancy among women using GLP‐1 receptor agonists.

## Methods

For our retrospective open cohort study, we analysed data for women aged 18–49 years who were considered active patients at general practices (three or more visits to the same practice during the study period^14^) during 1 January 2011 – 31 July 2022.

### Data source

We analysed data from the MedicineInsight dataset, a large national general practice dataset established by NPS MedicineWise;^15^ custodianship of MedicineInsight data was transferred to the Australian Commission on Safety and Quality in Health Care in 2023.^16^ MedicineInsight uses third party extraction tools (GRHANITE; cdmNet [Precedence Health Care]) to extract, de‐identify, and securely transmit patient data from the clinical information systems of participating practices, such as Best Practice and Medical Director, to a secure data repository. The extraction tool regularly collects incremental data, producing a longitudinal dataset in which individual patients at a practice can be tracked over time. The MedicineInsight dataset includes data on demographic characteristics, practice encounters (excluding progress notes), diagnoses, prescribed medications, and pathology tests, supplemented by selected free text information. In MedicineInsight, sex is categorised as female, male, or intersex/indeterminate. MedicineInsight contains electronic health records data from 662 general practices (8.2% of Australian practices) and more than 2700 general practitioners across Australia. The characteristics of active patients in MedicineInsight are nationally representative of all Australian general practice patients.^15^

### Outcomes

#### Use of GLP‐1 receptor agonists

We identified the first documented date of prescribing of GLP‐1 receptor agonists using World Health Organization Anatomic Therapeutic Chemical codes for exenatide (A10BJ01), liraglutide (A10BJ02), dulaglutide (A10BJ05), and semaglutide (A10BJ06).^17^ We included prescriptions issued by the general practitioners themselves, or those entered into a patient's medication history when prescribed by another medical practitioner (eg, an endocrinologist).

#### Overlap of GLP‐1 receptor agonist and contraceptive use

We determined the overlap of the use of GLP‐1 receptor agonists and of highly effective contraception methods: long acting reversible contraception (LARC; levonorgestrel intrauterine device [IUD], etonogestrel implant, copper IUD) or other (combined oral contraceptive pills, progestin‐only pills, depot injection, vaginal ring) (Supporting Information, table 1).

Contraception coverage was defined as a contraceptive method being prescribed prior to the prescribing of GLP‐1 receptor agonist and the estimated duration of contraceptive use or efficacy overlapping with the prescribing of the GLP‐1 receptor agonist. For LARC methods, contraceptive efficacy was defined as three years for implants, five years for hormonal IUDs, and ten years for copper IUDs. For shorter acting contraceptive methods, efficacy was defined according to the estimated duration of use, based on the contraceptive quantity prescribed (eg, for combined oral contraceptive pills, a four‐month supply is standard) and the number of repeats allowed (permitting a woman to receive multiple supplies using the same prescription). For example, a woman prescribed a four‐month supply of combined oral contraceptive pills with two repeats was estimated to have used the contraceptive for twelve months. We used information in clinical encounter and diagnosis records to identify the dates on which LARC devices were removed earlier than their estimated efficacy duration.

#### Pregnancy

Pregnancies within six months of being prescribed a GLP‐1 receptor agonist were identified by searching subsequent clinical encounters and documented diagnosis fields related to pregnancy, as well as prescribing records indicating the provision of medical abortion. The evaluation of pregnancies was limited to those for women with one or more clinical encounters after the first GLP‐1 receptor agonist prescription and for whom six months of follow‐up data were available (ie, the GLP‐1 receptor agonist was prescribed prior to 2022).

### Covariates

The patient characteristics included in analyses were age at time of GLP‐1 receptor agonist prescribing, concessional health care card status, smoking status, Indigenous status, residential remoteness, and residential socio‐economic status; other ethnic background information was not available. Age was categorised as 18–24, 25–29, 30–34, 35–39, 40–44, or 45–49 years. Women whose Indigenous status was recorded as unknown were categorised as non‐Indigenous, consistent with other studies.^18^ Remoteness and socio‐economic status were based on residential postcodes. Remoteness was defined according to the Australian Bureau of Statistics Australian Statistical Geography Standard (ASGS) remoteness areas classification;^19^ we combined data for women residing in inner regional and outer regional areas, and for those residing in remote and very remote areas. Socio‐economic status was defined according to the Index of Relative Socio‐Economic Advantage and Disadvantage (IRSAD),^20^ categorised as very low (deciles 1 or 2), low (3 or 4), middle (5 or 6), high (7 or 8), or very high (9 or 10). Whether women had diagnosed type 2 diabetes at the time of GLP‐1 receptor agonist prescribing was based on the date of diagnosis and validated diagnostic flags developed by MedicineInsight.^21^ We identified women with diagnosed polycystic ovary syndrome at the time of GLP‐1 receptor agonist prescribing. Body mass index (BMI), calculated from the most recent anthropometric data collected during the three months preceding GLP‐1 receptor agonist prescribing, was categorised as normal weight (18.5–24.9 kg/m^2^), overweight (25.0–29.9 kg/m^2^), obese class I (30.0–34.9 kg/m^2^), obese class II (35.0–39.9 kg/m^2^), or obese class III (40.0 kg/m^2^ or greater).

### Statistical analysis

We summarise patient characteristics as numbers and proportions or medians with interquartile ranges (IQRs). Women for whom some covariate data were missing were included in analyses without imputing the missing data. The annual incidence of GLP‐1 receptor agonist prescribing during 2011–2022 was separately calculated for women with and without diagnosed type 2 diabetes by dividing the number of women prescribed GLP‐1 receptor agonists by the total number who attended general practices. Incidence rates were directly age‐standardised against the 2001 Australian standard population.^22^ We report the characteristics of women prescribed GLP‐1 receptor agonists by type 2 diabetes status, contraception overlap, and documented pregnancy. Associations of outcomes with study covariates were evaluated using generalised linear models (Poisson distributions) with robust variance estimates; we report adjusted relative risks (aRRs) with 95% confidence intervals (CIs). Temporal changes in contraception overlap by diabetes status were plotted using three‐year moving means (two‐year means at the end values). Statistical analyses were performed in Stata MP 18.

### Ethics approval

The independent MedicineInsight Data Governance Committee approved the study (protocol 2019‐003), and the human research ethics committee of the University of Adelaide exempted our analysis of non‐identifiable data from formal ethics review.

## Results

Of 1 635 684 women aged 18–49 years included in the analysis, 18 010 (1.1%) were first prescribed GLP‐1 receptor agonists during 1 January 2011 – 31 July 2022; type 2 diabetes diagnoses were recorded for 3739 of these women (20.8%). The age‐standardised incidence of GLP‐1 receptor agonist prescribing for women with type 2 diabetes increased from 13.0 per 1000 women in 2011 to 88.5 per 1000 women in 2022; for women without type 2 diabetes, it increased from 0 to 14.9 per 1000 women (Box 1). Of the 6954 women first prescribed GLP‐1 receptor agonists during 2022, 6293 (90.5%) did not have diagnosed type 2 diabetes.

The median age (43 years; IQR, 37–47 years *v* 38 years; IQR, 31–44 years) and BMI (38.8 kg/m^2^; IQR, 33.8–45.1 kg/m^2^ *v* 35.2 kg/m^2^; IQR, 31.4–40.2 kg/m^2^) at the time of first GLP‐1 receptor agonist prescribing were higher for women diagnosed with type 2 diabetes than for those without diagnosed type 2 diabetes. Further, compared with women not diagnosed with type 2 diabetes, larger proportions of women with type 2 diabetes held health care concession cards (45.1% *v* 24.4%; aRR, 1.36; 95% CI, 1.27–1.45), were Indigenous women (11.1% *v* 5.2%; aRR, 1.27; 95% CI, 1.15–1.39), and had polycystic ovary syndrome (13.0% *v* 11.8%; aRR, 1.24; 95% CI, 1.13–1.36); a smaller proportion of women with type 2 diabetes had been prescribed GLP‐1 receptor agonists by general practitioners (89.0% *v* 94.3%; aRR, 0.73; 95% CI, 0.64–0.83) (Box 2).

Box 1 Incidence of first glucagon‐like peptide‐1 receptor agonist prescribing for women aged 18–49 years, 2011–2022, by type 2 diabetes status*

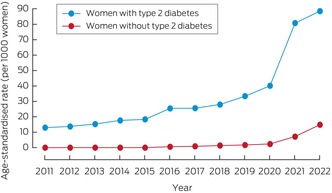

* Three‐year moving means (two‐year averages for the extremes).

Box 2 **Characteristics of women aged 18–49 years at the time of first prescribing of glucagon‐like peptide‐1 (GLP‐1) receptor agonists, 2011–2022, by type 2 diabetes status**

**Table:** 

Characteristic	Type 2 diabetes	No type 2 diabetes	Unadjusted relative risk (95% CI)	Adjusted relative risk* (95% CI)
Number of women	3739	14 271	—	—
Age group (years)				
18–24	94 (2.5%)	957 (6.7%)	0.68 (0.55–0.84)	0.64 (0.49–0.84)
25–29	188 (5.0%)	1769 (12.4%)	0.73 (0.62–0.86)	0.74 (0.61–0.89)
30–34	374 (10.0%)	2455 (17.2%)	1	1
35–39	619 (16.6%)	2903 (20.3%)	1.33 (1.18–1.50)	1.23 (1.08–1.40)
40–44	959 (25.6%)	3063 (21.5%)	1.80 (1.62–2.01)	1.50 (1.33–1.70)
45–49	1505 (40.3%)	3124 (21.9%)	2.46 (2.22–2.73)	1.78 (1.58–2.00)
Concession card holder	1687 (45.1%)	3475 (24.4%)	2.20 (1.94–2.16)	1.36 (1.27–1.45)
Smoking status				
Never smoked	1899 (54.2%)	8305 (64.2%)	1	1
Formerly smoked	924 (26.4%)	2889 (22.3%)	1.30 (1.21–1.40)	0.99 (0.92–1.06)
Currently smokes	682 (19.5%)	1737 (13.4%)	1.51 (1.40–1.63)	1.08 (0.99–1.17)
Missing data	234	1340	—	—
Remoteness^19^				
Major city	2063 (55.5%)	8750 (61.6%)	1	1
Inner/outer regional	1571 (42.3%)	5160 (36.3%)	1.22 (1.15–1.30)	0.97 (0.90–1.04)
Remote/very remote	81 (2.2%)	304 (2.1%)	1.10 (0.91–1.34)	0.96 (0.77–1.19)
Missing data	24	57	—	—
Socio‐economic status (IRSAD deciles)^20^				
Very low (1 or 2)	864 (23.3%)	2171 (15.3%)	1.78 (1.61–1.97)	1.09 (0.97–1.24)
Low (3 or 4)	857 (23.1%)	3026 (21.3%)	1.38 (1.25–1.53)	1.02 (0.91–1.15)
Middle (5 or 6)	877 (23.6%)	3463 (24.4%)	1.26 (1.14–1.40)	1.02 (0.90–1.15)
High (7 or 8)	646 (17.4%)	3082 (21.7%)	1.08 (0.97–1.21)	1.02 (0.90–1.15)
Very high (9 or 10)	471 (12.7%)	2472 (17.4%)	1	1
Indigenous status				
Aboriginal or Torres Strait Islander	414 (11.1%)	741 (5.2%)	1.82 (1.67–1.97)	1.27 (1.15–1.39)
Non‐Indigenous	3325 (88.9%)	13 530 (94.8%)	1	1
Body mass index category (kg/m^2^)				
Normal weight (18.5–24.9)	31 (1.4%)	118 (1.1%)	1.62 (1.16–2.27)	1.21 (0.93–1.56)
Overweight (25–29.9)	226 (10.5%)	1538 (14.9%)	1	1
Obesity class I (30–34.9)	408 (19.0%)	3340 (32.3%)	0.85 (0.73–0.99)	0.88 (0.78–1.00)
Obesity class II (35–39.9)	520 (24.2%)	2630 (25.5%)	1.29 (1.11–1.49)	1.15 (1.02–1.29)
Obesity class III (40 or greater)	964 (44.9%)	2707 (26.2%)	2.05 (1.79–2.34)	1.34 (1.20–1.50)
Missing data	1590	3938	—	—
Polycystic ovary syndrome	486 (13.0%)	1678 (11.8%)	1.09 (1.01–1.19)	1.24 (1.13–1.36)
Prescriber initiating GLP‐1 receptor agonist				
General practitioner	3329 (89.0%)	13 461 (94.3%)	0.59 (0.54–0.64)	0.73 (0.64–0.83)
Other	410 (11.0%)	810 (5.7%)	1	1
GLP‐1 receptor agonist				
Dulaglutide	737 (19.7%)	397 (2.8%)	4.73 (4.41–5.06)	3.65 (3.30–4.04)
Exenatide	1525 (40.8%)	218 (1.5%)	6.36 (6.01–6.73)	3.36 (2.95–3.82)
Liraglutide	327 (8.7%)	6442 (45.1%)	0.35 (0.31–0.40)	0.33 (0.29–0.39)
Semaglutide	1150 (30.8%)	7214 (50.6%)	1	1

CI = confidence interval; IRSAD = Index of Relative Socio‐economic Advantage and Disadvantage.

* Adjusted for all other covariates in the table and calendar year.

### Contraception overlap

Contraception overlap with first prescribing of GLP‐1 receptor agonists was determined for 3825 of 18 010 women (21.2%). The proportion of women with contraception overlap at the time of GLP‐1 receptor agonist prescribing was relatively stable between 2011 and 2022 (Box 3). The proportion was smaller among those with type 2 diabetes than among women without type 2 diabetes (16.6% *v* 22.5%; aRR, 0.88; 95% CI, 0.80–0.97). Contraception overlap was provided by non‐LARC methods for 1760 of 3204 women without type 2 diabetes (55%; 1511 using combined oral contraceptive pills) and 275 of 621 women with type 2 diabetes (44%; 237 using combined oral contraceptive pills) (Supporting Information, table 2). The relationship between individual characteristics and contraception overlap were similar for women with and without type 2 diabetes (Supporting Information, tables 3 and 4).

Box 3 **Contraception overlap among women aged 18–49 years at the time of first prescribing of glucagon‐like peptide‐1 (GLP‐1) receptor agonists, 2011–2022, by type 2 diabetes status**

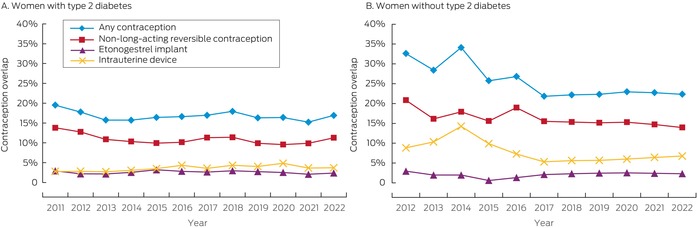

### Pregnancies within six months of GLP‐1 receptor agonist prescribing

At least six months of follow‐up data were available for 10 781 women; pregnancies within six months of the first prescribing of GLP‐1 receptor agonists were documented for 232 women (2.2%). Among women with type 2 diabetes, the pregnancy rate was highest among those aged 18–29 years (nine of 225 women; 4.0%); for women without type 2 diabetes, it was highest among women aged 30–34 years (76 of 1297 women; 5.9%) (Box 4). The characteristics of women who conceived within six months were similar to those of women who did not, except that the proportion with polycystic ovary syndrome was larger (27.2% *v* 11.7%; aRR, 2.04; 95% CI, 1.43–2.92) (Supporting Information, table 5). Contraception overlap at the time of GLP‐1 receptor agonist prescribing was associated with reduced risk of documented pregnancy (1.7% *v* 2.3%; aRR, 0.62; 95% CI, 0.41–0.94).

Box 4 **Proportion of women aged 18–49 years who conceived within six months of the first prescribing of glucagon‐like peptide‐1 (GLP‐1) receptor agonists, 2011–2022, by type 2 diabetes status**

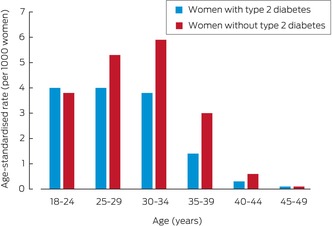

## Discussion

In our large cohort study, we found that prescribing of GLP‐1 receptor agonists for women of reproductive age attending Australian general practices increased during 2015–2022, and particularly rapidly during 2020–2022; we also found that the level of contraception coverage at the time of treatment initiation was low (below 25%). Initial prescribing of GLP‐1 receptor agonists increased among women with or without type 2 diabetes, which suggests increasing off‐label use for indications such as weight loss. Further, pregnancies within six months of initial GLP‐1 receptor agonist prescribing are not infrequent (2.2% of women).

The increase in GLP‐1 receptor agonist prescribing we found mirrors that of its increased overall prescribing in Australia;^23^ the rapid increase since 2020 corresponds to the regulatory approval of semaglutide and its inclusion in the Pharmaceutical Benefits Scheme (PBS) in July 2020.^24^ A recent evaluation of PBS claims data found that about 120 000 Australians were dispensed GLP‐1 receptor agonists during July 2022, but the number of women of reproductive age was not reported, and off‐label prescribing could not be assessed.^23^ Despite being licensed and subsidised only for managing type 2 diabetes,^24^ in our analysis the absolute increase in GLP‐1 receptor agonist prescribing was larger among women without type 2 diabetes. Similar findings were reported by recent cohort studies in Norway and Denmark.^25^, ^26^ Given that GLP‐1 receptor agonists are increasingly prescribed for off‐label uses such as weight loss, as well as high rates of overweight and obesity among women of reproductive age, clinical practice guidelines are needed to support their safe and effective use in women in this age group.

The low levels of highly effective contraception coverage among women commencing treatment with GLP‐1 receptor agonist is concerning. The overall rates of LARC use when commencing GLP‐1 receptor agonist treatment in our study was 6.3% for women with type 2 diabetes and 8.7% for those without type 2 diabetes, lower than the rate for all Australian women in 2018 (10.8%).^27^ Lower contraception coverage among women with type 2 diabetes is particularly concerning because of their greater need for pre‐conception care and increased risk of adverse pregnancy outcomes.^28^ The reasons for the low coverage level could be related to limited awareness of the risks associated with GLP‐1 receptor agonist use during pregnancy, or perceptions of reduced fertility in women with type 2 diabetes, polycystic ovary syndrome, or obesity.^29^ However, as modest reductions in weight can improve fertility,^30^ the risk of unintended pregnancy is significant if effective contraception is not used. Conversely, the use of GLP‐1 receptor agonists for improving fertility has attracted interest, but even during intended pregnancies their use entails risks.^31^ Further, GLP‐1 receptor agonists may reduce the effectiveness of oral contraception by altering drug absorption,^32^ but the clinical significance of this interaction was questioned in a recent systematic review.^33^

Information about the safety of GLP‐1 receptor agonists during pregnancy is limited. In animal studies, exposure to GLP‐1 receptor agonists leads to reduced fetal weight and growth, altered ossification, and congenital visceral and skeletal malformations.^11^ As these effects were coupled with significant maternal weight loss, they raise questions about whether the changes were directly related to GLP‐1 receptor agonists or were indirect consequences of maternal weight loss. The few human studies provide some reassurance about their safety during pregnancy.^34^, ^35^ A recent cohort study (938 pregnancies in women using GLP‐1 receptor agonists) found no significant difference in the adjusted risk of major congenital malformations compared with women receiving insulin (adjusted risk ratio, 0.95; 95% CI, 0.72–1.26), but confounding by differences in glucose control were possible.^12^ Supporting these reassuring findings is evidence that the placental transfer of GLP‐receptor agonists is limited.^36^, ^37^ However, given the metabolic programming effects of GLP‐1 receptor agonists,^38^ safety concerns are not limited to the risk of major congenital malformations. Concerns about short and long term effects on fetal growth and metabolic health are related to reports of increased risk of small for gestational age babies among women who lose weight while pregnant,^39^ indicating that rapid weight loss itself, immediately prior to or during early pregnancy, might increase the risk of adverse pregnancy outcomes.

### Limitations

We analysed data from a large, broadly nationally representative general practice dataset.^15^ Further, the longitudinal dataset includes data on all GLP‐1 receptor agonist prescriptions issued by or recorded by participating general practices, irrespective of indication, as well as detailed information about medical conditions at the individual level. However, the dataset covers only about 8% of all Australian general practices and may not be nationally representative, as participating practices are self‐enrolled rather than randomly selected. GLP‐1 receptor agonist prescriptions data were available, but we could not determine the proportion of women to whom the medication was dispensed, potentially leading to overestimation of use. Contraception overlap was based on estimated duration of efficacy, but some women may have ceased contraception before using GLP‐1 receptor agonists, leading to overestimation of contraception overlap. As patient records are not linked across different general practices and hospitals, data related to encounters outside an individual's usual general practice may be incomplete. Duplication of patient information is possible if women attended multiple MedicineInsight sites, but the estimated duplication rate is about 4%.^15^ We may have underestimated number of pregnancies within six months of the first GLP‐1 receptor agonist prescription, as we relied on general practice data documentation. Data for the outcomes of documented pregnancies were not available.

### Conclusion

The prescribing of GLP‐1 receptor agonists is rapidly increasing among women of reproductive age, and they are most frequently prescribed for indications other than glucose management in people with type 2 diabetes. Concurrent highly effective contraception use at the time of treatment initiation is low, and a substantial number of women are pregnant within six months of commencing GLP‐1 receptor agonist. This raises concerns about potential harms resulting from unintended pregnancies among women using GLP‐1 receptor agonists. Our findings indicate that further evidence and guidelines are needed to support the safe and effective use of GLP‐1 receptor agonists by women of reproductive age. Clearer practice recommendations are not only needed for women with type 2 diabetes, but also for those with polycystic ovary syndrome or obesity, with appropriate emphasis on ensuring concurrent contraception.

## Open access

Open access publishing facilitated by Flinders University, as part of the Wiley – Flinders University agreement via the Council of Australian University Librarians.

## Competing interests

No relevant disclosures.

## Data sharing

This study did not generate any original data.

## Author contributions

Kailash Thapaliya: conceptualisation; methodology; formal analysis; writing (original draft). Arianne Sweeting: writing (review and editing). Kirsten Black: writing (review and editing). Amanda Poprzeczny: writing (review and editing). Danielle Mazza: writing (review and editing). Luke Grzeskowiak: conceptualisation; formal analysis; methodology; writing (review and editing).

Received 9 July 2024, accepted 20 May 2025

## Supporting information


**Data S1** Supplementary methods
