# Supplementary material for: Incidence of GLP‐1 receptor agonist use by women of reproductive age attending general practices in Australia, 2011–2022: a retrospective open cohort study
Source: Med J Aust. 2025 Sep 1;223(7):365–71. doi: 10.5694/mja2.70026 (PMC12502889; doi:10.5694/mja2.70026)
Supplement: Supplementary file 1 — Data S1 Supplementary methods [file MJA2-223-365-s001.pdf]

MJA
The Medical Journal of Australia

Supporting Information

Supplementary methods and results

**This appendix was part of the submitted manuscript and has been peer reviewed.
It is posted as supplied by the authors.**

Appendix to: Thapaliya K, Sweeting A, Black KI, et al. Incidence of GLP-1 receptor agonist use by women of reproductive age attending general practices in Australia, 2011–2022: a retrospective open cohort study. Med J Aust 2025; doi: 10.5694/mja2.70026.

Supplementary methods

Table 1. Contraceptives and their Anatomic Therapeutic Category (ATC) codes

Category	Names	Medication	ATC codes
**Long acting reversible contraceptives**	Levonorgestrel IUD	Levonorgestrel	G02BA03
	Etonogestrel Implant	Etonogestrel	G03AC08
	Copper IUD	Plastic IUD with copper	G02BA02
**Short acting reversible contraceptives**	Combined hormonal contraceptives	*Monophasic combinations*	
		Drospirenone with ethinylestradiol	G03AA12
		Levonorgestrel with enthinylestradiol	G03AA07
		Norethindrone and ethinyl estradiol	G03AA05
		Desogestrel with ethinylestradiol	G03AA09
		Drospirenone and ethinylestradiol	G03AA18
		Levonorgestrel and ethinylestradiol	G03AA07
		Norgesteril and ethinylestradiol	G03AA06
		Drospirenone and estetrol	G03AA12
		Nomegestrol and estradiol	G03AA14
		Cyproterone with ethinylestradiol	G03HB01
		Multiphasic combinations	
		Dienogest and estradiol	G03AB08
		Norethindrone and ethinylestradiol	G03AB04
		Desogestrel and ethinylestradiol	G03AB05
		Levonorgestrel and ethinyl estradiol	G03AB03
	Progestins only pills	Norethisterone	G03AC01
		Drospirenone	G03AC10
		Levonorgestrel	G03AC03
		Norethisterone	G03AC06
	Depot injection	Medroxyprogesterone (Suspension)	G03AC06
	Vaginal ring	Etonogestrel and ethinylestradiol	G02BB01

IUD = intrauterine device.

Supplementary results

Table 2. Type of contraceptive overlap among women aged 18–49 years first prescribed glucagon-like peptide-1 (GLP-1) receptor agonists, 2011–2022, by type 2 diabetes status

	Type 2 diabetes		Unadjusted relative risk (95% CI)	Adjusted relative risk (95% CI)*
**Contraceptive type**	**Yes**	**No**		
**Number of women**	**3739**	**14 271**		
**Any contraceptive**	621 (16.6%)	3204 (22.5%)	0.74 (0.68-0.80)	0.88 (0.80-0.97)
**Any long-acting reversible contraceptive**	237 (6.3%)	1244 (8.7%)	0.76 (0.67-0.85)	0.82 (0.70-0.95)
Hormonal intrauterine device	138 (3.7%)	877 (6.1%)	0.64 (0.55-0.75)	0.66 (0.54-0.81)
Implants	94 (2.5%)	326 (2.3%)	1.08 (0.90-1.29)	1.14 (0.93-1.41)
Copper intrauterine device	5 (0.1%)	44 (0.3%)	0.49 (0.21-1.13)	1.05 (0.46-2.40)
**Any non-long-acting reversible contraceptive**	275 (7.4%)	1760 (12.3%)	0.62 (0.56-0.70)	0.80 (0.69-0.92)
Combined oral contraceptive pill	237 (6.3%)	1511 (10.6%)	0.63 (0.56-0.71)	0.78 (0.67-0.91)
Depo injection	124 (3.3%)	292 (2.0%)	1.45 (1.25-1.69)	1.36 (1.13-1.62)
Progestogen only pill	38 (1.0%)	252 (1.8%)	0.63 (0.47-0.84)	0.91 (0.68-1.23)
Vaginal ring	1 (0.0%)	21 (0.1%)	0.22 (0.03-1.48)	0.82 (0.14-4.83)

CI = confidence interval.

* Adjusted for age, concessional status, smoking status, remoteness, socio-economic status, Indigenous status, body mass index, polycystic ovarian syndrome, prescriber type initiating GLP-1 receptor agonist treatment, and calendar year.

Table 3. Contraception overlap among women aged 18–49 years with type 2 diabetes first prescribed glucagon-like peptide-1 (GLP-1) receptor agonists, 2011–2022, by characteristic

Characteristic	Contraceptive overlap	No contraceptive overlap	Unadjusted Relative Risk (95%CI)	Adjusted Relative risk† (95% CI)
**Number of women**	**621**	**3118**	—	—
**Age group (years)**				
18-24	17 (2.7%)	77 (2.5%)	0.91 (0.57-1.47)	0.96 (0.50-1.85)
25-29	46 (7.4%)	142 (4.6%)	1.24 (0.89-1.71)	1.25 (0.81-1.92)
30-34	74 (11.9%)	300 (9.6%)	1	1
35-39	129 (20.8%)	490 (15.7%)	1.05 (0.82-1.36)	1.11 (0.80-1.54)
40-44	173 (27.9%)	786 (25.2%)	0.91 (0.71-1.16)	0.83 (0.60-1.15)
45-49	182 (29.3%)	1323 (42.4%)	0.61 (0.48-0.78)	0.57 (0.41-0.78)
**Concession card holder**	286 (46.1%)	1401 (44.9%)	1.04 (0.90-1.20)	0.99 (0.80-1.22)
**Smoking status**				
Never smoked	342 (59.0%)	1557 (53.2%)	1	1
Formerly smoked	145 (25.0%)	779 (26.6%)	0.87 (0.73-1.04)	0.85 (0.68-1.08)
Currently smokes	93 (16.0%)	589 (20.1%)	0.76 (0.61-0.94)	0.78 (0.59-1.04)
Missing data	41	193	—	—
**Remoteness**				
Major city	310 (50.3%)	1753 (56.6%)	1	1
Inner/outer regional	296 (48.1%)	1275 (41.1%)	1.25 (1.08-1.45)	1.43 (0.15-1.79)
Remote/very remote	10 (1.6%)	71 (2.3%)	0.82 (0.46-1.48)	1.20 (0.62-2.34)
Missing data	5	19	—	—
**Socio-economic status (IRSAD deciles)**				
Very low (1 or 2)	142 (23.1%)	722 (23.3%)	0.90 (0.71-1.15)	0.73 (0.50-1.07)
Low (3 or 4)	144 (23.4%)	713 (23.0%)	0.92 (0.72-1.17)	0.85 (0.59-1.22)
Middle (5 or 6)	142 (23.1%)	735 (23.7%)	0.89 (0.70-1.13)	0.84 (0.58-1.21)
High (7 or 8)	102 (16.6%)	544 (17.6%)	0.86 (0.67-1.12)	0.92 (0.64-1.33)
Very high (9 or 10)	86 (14.0%)	385 (12.4%)	1	1
**Indigenous status**				
Aboriginal or Torres Strait Islander	66 (10.6%)	348 (11.2%)	0.98 (0.76-1.21)	1.03 (0.77-1.39)
Non-Indigenous	555 (89.4%)	2770 (88.8%)	1	1
**Body mass index (kg/m^2)**				
Normal weight (18.5-24.9)	5 (1.4%)	26 (1.5%)	0.83 (0.36-1.93)	0.91 (0.38-2.18)
Overweight (25-29.9)	44 (12.1%)	182 (10.2%)	1	1
Obesity class I (30-34.9)	73 (20.0%)	335 (18.8%)	0.92 (0.66-1.29)	0.94 (0.67-1.33)
Obesity class II (35-39.9)	81 (22.2%)	439 (24.6%)	0.80 (0.57-1.12)	0.81 (0.57-1.14)
Obesity class III (≥40)	162 (44.4%)	802 (45.0%)	0.86 (0.64-1.17)	0.86 (0.63-1.19)
Missing data	256	1334	—	—
**Polycystic ovary syndrome**	82 (13.2%)	404 (13.0%)	1.02 (0.82-1.26)	0.77 (0.58-1.03)
**Prescriber initiating GLP-1 receptor agonist**				
General practitioner	563 (90.7%)	2766 (88.7%)	1.20 (0.93-1.54)	1.33 (0.85-2.08)
Other	58 (9.3%)	352 (11.3%)	1	1

CI = confidence interval.

* Adjusted for all other covariates in the table and calendar year.

Table 4. Contraception overlap among women aged 18–49 years without type 2 diabetes first prescribed glucagon-like peptide-1 (GLP-1) receptor agonists, 2011–2022, by characteristic

Characteristic	Contraceptive overlap	No contraceptive overlap	Unadjusted relative risk (95%CI)	Adjusted relative risk† (95% CI)
**Number of women**	**3204**	**11 067**	—	—
**Age group (years)**				
18-24	333 (10.4%)	624 (5.6%)	1.42 (1.27-1.58)	1.49 (1.30-1.70)
25-29	542 (16.9%)	1227 (11.1%)	1.25 (1.13-1.38)	1.26 (1.12-1.42)
30-34	603 (18.8%)	1852 (16.7%)	1	1
35-39	679 (21.2%)	2224 (20.1%)	0.95 (0.87-1.05)	0.98 (0.87-1.10)
40-44	590 (18.4%)	2473 (22.3%)	0.78 (0.71-0.87)	0.79 (0.70-0.90)
45-49	457 (14.3%)	2667 (24.1%)	0.60 (0.53-0.66)	0.58 (0.50-0.66)
**Concession card holder**	785 (24.5%)	2690 (24.3%)	1.01 (0.94-1.08)	0.98 (0.90-1.07)
**Smoking status**				
Never smoked	1993 (66.8%)	6312 (63.5%)	1	1
Formerly smoked	633 (21.2%)	2256 (22.7%)	0.91 (0.84-0.99)	1.00 (0.91-1.10)
Currently smokes	359 (12.0%)	1378 (13.9%)	0.86 (0.78-0.95)	0.91 (0.81-1.02)
Missing data	1121	219	—	—
**Remoteness**				
Major city	1884 (59.1%)	6866 (62.3%)	1	1
Inner/outer regional	1254 (39.3%)	3906 (35.4%)	1.13 (1.06-1.20)	1.06 (0.98-1.16)
Remote/very remote	52 (1.6%)	252 (2.3%)	0.79 (0.62-1.02)	0.79 (0.59-1.08)
Missing data	14	43	—	—
**Socio-economic status (IRSAD deciles)**				
Very low (1 or 2)	503 (15.8%)	1668 (15.1%)	1.18 (1.06-1.32)	1.14 (0.99-1.32)
Low (3 or 4)	748 (23.4%)	2278 (20.7%)	1.26 (1.14-1.39)	1.23 (1.08-1.40)
Middle (5 or 6)	804 (25.2%)	2659 (24.1%)	1.18 (1.07-1.31)	1.16 (1.02-1.32)
High (7 or 8)	649 (20.3%)	2433 (22.1%)	1.07 (0.96-1.19)	1.06 (0.93-1.21)
Very high (9 or 10)	486 (15.2%)	1986 (18.0%)	1	1
**Indigenous status**				
Aboriginal or Torres Strait Islander	163 (5.1%)	578 (5.2%)	0.98 (0.85-1.12)	0.87 (0.74-1.03)
Non-Indigenous	3041 (94.9%)	10489 (94.8%)	1	1
**Body mass index (kg/m^2)**				
Normal weight (18.5-24.9)	22 (1.0%)	96 (1.2%)	0.78 (0.53-1.14)	0.77 (0.51-1.14)
Overweight (25-29.9)	369 (16.0%)	1169 (14.6%)	1	1
Obesity class I (30-34.9)	751 (32.6%)	2589 (32.2%)	0.94 (0.84-1.05)	0.91 (0.81-1.01)
Obesity class II (35-39.9)	593 (25.7%)	2037 (25.4%)	0.94 (0.84-1.05)	0.88 (0.79-0.99)
Obesity class III (≥40)	568 (24.7%)	2139 (26.6%)	0.87 (0.78-0.98)	0.82 (0.73-0.92)
Missing data	901	3037	—	—
**Polycystic ovary syndrome**	380 (11.9%)	1298 (11.7%)	1.01 (0.92-1.11)	0.88 (0.78-0.99)
**Prescriber initiating GLP-1 receptor agonist**				
General practitioner	3038 (94.8%)	10423 (94.2%)	1.10 (0.96-1.27)	1.06 (0.85-1.32)
Other	166 (5.2%)	644 (5.8%)	1	1

CI = confidence interval.
* Adjusted for all other covariates in the table and calendar year.

Table 5. Documented pregnancy within six months of initial prescribing of glucagon-like peptide-1 (GLP-1) receptor agonists for women aged 18–49 years, 2011–2022, by characteristic

Characteristics	Pregnancy	No pregnancy	Unadjusted Relative Risk (95%CI)	Adjusted Relative risk† (95% CI)
**Number of women**	**232**	**10 549**	—	—
**Age group (years)**				
18-24	22 (9.5%)	557 (5.3%)	0.69 (0.44-1.09)	0.81 (0.47-1.39)
25-29	55 (23.7%)	1022 (9.7%)	0.93 (0.67-1.29)	0.86 (0.56-1.32)
30-34	87 (37.5%)	1497 (14.2%)	1	1
35-39	54 (23.3%)	2016 (19.1%)	0.47 (0.34-0.66)	0.52 (0.34-0.80)
40-44	12 (5.2%)	2439 (23.1%)	0.09 (0.05-0.16)	0.13 (0.06-0.26)
45-49	2 (0.9%)	3018 (28.6%)	0.01 (0.00-0.05)	0.01 (0.00-0.09)
**Concession card holder**	72 (31.0%)	3242 (30.7%)	1.01 (0.77-1.34)	1.00 (0.68-1.46)
**Smoking status**				
Never smoked	145 (67.1%)	5837 (60.0%)	1	1
Formerly smoked	36 (16.7%)	2390 (24.6%)	0.61 (0.43-0.88)	0.92 (0.60-1.42)
Currently smokes	35 (16.2%)	1499 (15.4%)	0.94 (0.65-1.36)	1.06 (0.67-1.69)
Missing data	16	823	—	—
**Remoteness**				
Major city	130 (56.3%)	6238 (59.4%)	1	1
Inner/outer regional	98 (42.4%)	4078 (38.8%)	1.15 (0.89-1.49)	1.08 (0.72-1.61)
Remote/very remote	3 (1.3%)	192 (1.8%)	0.75 (0.24-2.35)	0.39 (0.06-2.72)
Missing data	1	41	—	—
**Socio-economic status (IRSAD deciles)**				
Very low (1 or 2)	45 (19.5%)	1861 (17.7%)	1.52 (0.95-2.43)	0.89 (0.44-1.79)
Low (3 or 4)	54 (23.4%)	2289 (21.8%)	1.48 (0.94-2.33)	1.22 (0.66-2.24)
Middle (5 or 6)	59 (25.5%)	2466 (23.5%)	1.50 (0.96-2.35)	1.10 (0.61-1.96)
High (7 or 8)	45 (19.5%)	2117 (20.1%)	1.34 (0.84-2.14)	1.24 (0.71-2.15)
Very high (9 or 10)	28 (12.1%)	1775 (16.9%)	1	1
**Indigenous status**				
Aboriginal or Torres Strait Islander	19 (8.2%)	727 (6.9%)	1.20 (0.75-1.19)	1.26 (0.70-2.24)
Non-Indigenous	213 (91.8%)	9822 (93.1%)	1	1
**Body mass index (kg/m^2)**				
Normal weight (18.5-24.9)	2 (1.3%)	97 (1.4%)	1.03 (0.24-4.33)	1.00 (0.25-4.05)
Overweight (25-29.9)	20 (12.7%)	996 (14.0%)	1	1
Obesity class I (30-34.9)	47 (29.7%)	2032 (28.5%)	1.15 (0.68-1.93)	1.13 (0.67-1.91)
Obesity class II (35-39.9)	48 (30.4%)	1754 (24.6%)	1.35 (0.81-2.27)	1.17 (0.69-2.00)
Obesity class III (≥40)	41 (25.9%)	2241 (31.5%)	0.91 (0.54-1.55)	0.81 (0.47-1.41)
Missing data	74	3429	—	—
**Type 2 diabetes**	30 (12.9%)	3004 (28.5%)	0.38 (0.26-0.56)	0.65 (0.39-1.09)
**Polycystic ovary syndrome**	63 (27.2%)	1238 (11.7%)	2.72 (2.05-3.61)	2.04 (1.43-2.92)
**Prescriber initiating GLP-1 receptor agonist**				
General Practitioner	213 (91.8%)	9708 (92.0%)	0.97 (0.61-1.55)	0.88 (0.45-1.74)
Other	19 (8.2%)	841 (8.0%)	1	1

CI = confidence interval.
* Adjusted for all other covariates in the table and calendar year.
